# Sex Differences in Circulating Inflammatory, Immune, and Tissue Growth Markers Associated with Fabry Disease-Related Cardiomyopathy

**DOI:** 10.3390/cells14050322

**Published:** 2025-02-20

**Authors:** Margarita M. Ivanova, Julia Dao, Andrew Friedman, Neil Kasaci, Ozlem Goker-Alpan

**Affiliations:** Lysosomal and Rare Disorders Research and Treatment Center, Fairfax, VA 22030, USAogoker-alpan@ldrtc.org (O.G.-A.)

**Keywords:** Fabry disease, inflammation, cardio, biomarkers, sex, fibrosis, cytokines, genetics

## Abstract

Fabry disease (FD) is a lysosomal disorder due to alpha-galactosidase-A enzyme deficiency, accumulation of globotriaosylceramide (Gb3) and globotriaosylsphingosine (lyso-Gb3) which lead to proinflammatory effects. Males develop progressive hypertrophic cardiomyopathy (HCM) followed by fibrosis; females develop nonconcentric hypertrophy and/or early fibrosis. The inflammatory response to Gb3/lyso-Gb-3 accumulation is one of the suggested pathogenic mechanisms in FD cardiomyopathy when the secretion of inflammatory and transforming growth factors with infiltration of lymphocytes and macrophages into tissue promotes cardiofibrosis. This study aims to evaluate inflammation-driving cytokines and cardio-hypertrophic remodeling biomarkers contributing to sex-specific HCM progression. Biomarkers were studied in 20 healthy subjects and 45 FD patients. IL-2, IL-10, TNF-α, and IFN-γ were elevated in all patients, while IL-1α, MCP-1, and TNFR2 showed sex-specific differences. The increased cytokines were associated with the NF-kB pathway in FD males with HCM, revealing a correlation between MCP-1, IFN-γ, VEGF, GM-CSF, IL-10, and IL-2. In female patients, the impaired TNFα/TNFR2/TGFβ cluster with correlations to MCP-1, VEGF, GM-CSF, and IL-1α was observed. The activation of cytokines and the NF-kB pathway indicates significant inflammation during HCM remodeling in FD males. The TNFα/TNFR2/TGFβ signaling cluster may explain early fibrosis in females with FD cardiomyopathy. Sex-specific inflammatory responses in FD influence the severity and progression of HCM.

## 1. Introduction

Fabry disease (FD) is an inherited X-linked disorder characterized by a deficiency of the lysosomal enzyme α-galactosidase A (α–Gal A) (EC entry 3.2.1.22) due to mutations in the *GLA* gene (Gene Entrez: 2717; NCBI reference: NM_000169.3; OMIM #3006440). As *GLA* is carried on the X chromosome, hemizygous males present with more severe and early-onset symptoms, including cardiovascular diseases; renal failure; cerebrovascular complications, such as ischemic or hemorrhagic strokes; ocular and hearing complications; and neurologic complications [1,2,3]. Females in which the mutated X is inactivated, favoring the normal allele, could present with fewer symptoms; however, if the mutated allele is predominantly activated, females may experience severe symptoms, especially with advancing age [4,5]. As a result, females often present with a more unpredictable clinical course, including the degree and progression of the vital organ involvement such as heart, kidney, or brain. Clinically, FD presents two clinical types: classic and non-classic (late-onset form). The classical form presents with absent or severely deficient levels of a-Gal-A enzyme activity, leading to early disease onset. The residual a-Gal-A enzyme activity leads to non-classical FD that manifests later in life and often involves the heart [6].

α–Gal A catalyzes the lysosomal hydrolysis of globotriaosylceramide (Gb-3) to lactosylceramide and digalactosylceramide to galactosylceramide [7]. The deficiency of the α–Gal A enzyme leads to an accumulation of Gb-3 and its metabolite, known as globotriaosylsphingosine (Lyso-Gb-3), in the affected cells [8]. The deposition of Gb-3 and Lyso-Gb-3 within the myocardium affects cardiac function and results in progressive cardiovascular pathology [9]. Gb-3 accumulation has been found in the cardiac valves, cardiomyocytes, nerves, and coronary arteries. At the cellular level, Gb-3 and Lyso-Gb-3 accumulation triggers a cascade of events leading to inflammation and end-stage fibrosis [10,11]. Moreover, Lyso-Gb-3 deposition is associated with the increased release of inflammatory molecules and transforming growth factors related to myofibrosis [11,12]. Furthermore, circulated Lyso-GB3 in plasma blood and extracellular fluids may have a secondary toxic effect on cells, such as activating Ca^2+^ uptake of voltage-dependent nociceptive neurons and allodynia [13]. Since the voltage-gated Ca^2+^ channel is responsible for initiating muscle excitation–contraction coupling, it is possible that dysfunction in the Ca2+ channel could occur in other tissues as well. Endomyocardial biopsies reveal the infiltration of lymphocytes and macrophages in heart tissue, indicating that inflammation plays a significant role in cardiac damage. Several studies of patients with FD have shown increasing levels of circulated inflammatory cytokines, including Il-6, MCP-1, and TNF-α [10,14]. These findings suggest that the chronic presence of proinflammatory cytokines may actively contribute to the progression of FD [14]. The elevation of proinflammatory cytokines (NF-kB, IL-1α, IL-1β, TNFR2, TNF-α) in patients with chronic heart failure in general (without FD) and animal models further support our hypothesis that these inflammatory markers could be utilized for screening of cardiac involvement in asymptomatic patients with FD [15,16,17,18].

In patients with FD, cardiovascular complications are most frequently encountered, contributing substantially not only to morbidity but also are the leading cause of premature death in both genders. The manifestations of cardiac involvement in FD are left ventricular hypertrophy (LVH), diastolic dysfunction, and microvascular angina. However, the most common cardiac pathology observed in patients with FD is hypertrophic cardiomyopathy (HCM), which is also a leading cause of death [19].

Biological sex plays an essential role in the cardiovascular physiology and clinical presentation of heart diseases [20]. HCM is primarily genetic in etiology and affects 1 in 200–500 individuals in the general population. Women often receive a delayed initial evaluation and are diagnosed later than men. They commonly have chest pain and exertional dyspnea [21,22]. In patients with FD, males are affected more frequently than females at an early age. In contrast to male patients, FD females develop LVH approximately ten years later. However, in female patients with FD, the loss of cardiac function and the development of fibrosis may occur independently of myocardial hypertrophy. Therefore, cardiac monitoring and disease staging in these patients should focus on both detecting left ventricular (LV) hypertrophy and assessing for fibrosis [23].

This comprehensive research study aims to evaluate the impact of inflammation-driving cytokines and cardio-hypertrophic remodeling biomarkers on the progression of HCM specific to each sex in patients with FD. This investigation’s results could pave the way for developing personalized treatment approaches tailored to address FD-related cardiomyopathy based on gender, marking a significant intersection of cardiology and genetics.

## 2. Materials and Methods

### 2.1. Subjects

In an IRB-approved protocol # NCT04724083, 45 patients with FD (21 males and 24 females) (mean age: 40 ± 14 years) and 20 healthy controls (10 males and 10 females) (mean age: 48 ± 11 years) were studied. Study participants were categorized into two cohorts: FD with HCM (*n* = 28, mean age 44 ± 18) and without HCM (*n* = 17, mean age 35.1 ± 13) (Supplementary Table S1). The “borderline” group represents patients with an abnormal EKG (*n* = 6) (three females and three males); however, during the majority analysis, this group was included without the HCM cohort. All male patients with FD received enzyme replacement therapy (ERT), while only 9 female patients with HCM were on ERT. Three female subjects received chaperone therapy, one switched from ERT to chaperone therapy, and two were not treated (naïve). The values for GB3 and lyso-Gb3, as well as other relevant clinical information, were obtained from the patients’ medical records. Demographics, *GLA* genotypes, and other clinical and laboratory characteristics of patients with FD were described previously [12].

### 2.2. Sample Collection and Storage

Venous blood samples were collected in EDTA vacutainer tubes. After collection, blood was centrifuged for 5 min at 5000× *g*. Then, plasma was collected and aliquoted in small volumes in sterile tubes and stored at −80 °C before analysis.

### 2.3. Enzyme-Linked Immunosorbent Assay (ELISA)

As previously described, venous blood samples were collected in EDTA vacutainer tubes [18]. Plasma levels of inflammatory and growth factors biomarkers were measured using commercially available ELISA kits: MSP-1, INF-γ, FGF2, TNFR2, VEGF, TGF-β, IGF-1, Il-10, and Il-2 ELISA kits (Origene Technologies Inc, Rockville, MD, USA). The TNF-α and Il-6 concentrations were measured using the ELISA kit (Abcam, Cambridge, UK), and NF-kB and PIGF were purchased from MyBioSource (MyBioSource, Inc., Vancouver, BC, Canada). TNF-α, active TGF-β, IL-1β (Biolegend, San Diego, CA, USA), IL-1α (AntiBodies Online, Pottstown, PA, USA), and GM-CSF (Thermo Fisher Scientific, Rockville, MD, USA) were used to measure biomarkers in plasma using manufacturing protocols.

### 2.4. Statistical Analysis

Statistical analysis was performed using Graph Prizm (GraphPad 10, San Diego, CA, USA). Differences between the two groups were tested by Student’s *t*-test (unpaired, two-tailed) or an F-test to compare the variances. The groups were compared using a one-way analysis of variance (ANOVA) followed by Kruskal–Wallis tests. Integrative matrix analysis, using the Pearson or Spearman correlation technique, determined the correlation between biomarkers and clinical biomarkers (plasma Lyso-GB3 and urine Gb3). The correlation matrix was computed between pairs of columns. The data were sampled from a Gaussian distribution, and Pearson correlation coefficients were calculated. The results were visualized as a heatmap of the correlation matrix, or as scatter plots between two or three variables, with a 95% confidence interval. Multivariate statistical analysis, including principle component analysis (PCA), was used for PC-1 and PC-2 cluster plots.

## 3. Results

### 3.1. Early Onset of HCM Is Frequently Encountered in Female Patients with FD

**Figure 1:**
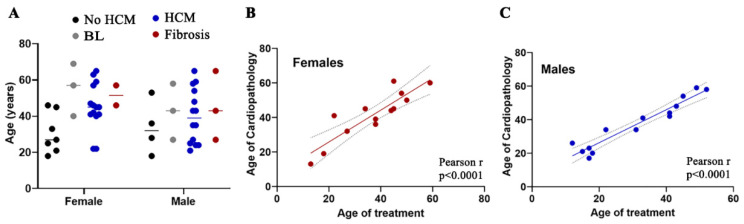
Onset diagnostic of hypertrophic cardiomyopathy (HCM) and relationship with enzyme replacement therapy (ERT) initiation in patients with FD. (**A**) The age range of female and male patients diagnosed with FD, with or without HCM, is categorized by individual values. BL—border line, patients without cardiomyopathy, but with abnormal EKG. (**B**,**C**) Correlation analysis between age at HCM diagnosis and ERT treatment in female (**B**) and male (**C**) patients with FD. Pearson correlation analysis is presented in the bottom right corner. BL (borderline), patients without HCM, but not normal EKG.

In the general population, women with HCM are diagnosed at an older age than men (females 59 ± 16 vs. males 52 ± 15 years), with the age difference ranging from 6 to 13 years [24,25]. Moreover, females are more likely to exhibit systemic hypertension prior to an HCM diagnosis and often show more echocardiographic evidence of diastolic dysfunction and obstruction, despite having similar left ventricular mass indices to men. In FD, it is a general assumption that the clinical presentation is not only milder in women at any age, but cardiac, cerebrovascular, and renal diseases present at least a decade later than in men [26,27]. However, it is important to note that this may not always be the case. In our cohort, the average age of females with a mild form of HCM was 43.6 ± 13 years; with a severe form of HCM, it was 50 ± 13 years (Supplementary Table S1 and Figure 1A). For males with FD, the average age with a mild form of HCM was 32 ± 12 years, and for a severe form of HCM, 47 ± 14 years [12]. Thus, like male FD patients, age is not a determining factor for predicting mild or severe forms of HCM in female patients (Supplementary Table S1). In some cases, young female patients with FD may exhibit severe cardiac pathology. For example, in our cohort, the youngest female with a mild form of HCM was 22 years old, whereas the youngest male with HCM was 21 years old (Figure 1A). Thus, the early detection of HCM in females with FD is crucial for effective disease management, as cardiac fibrosis can occur in females with FD even before hypertrophic changes are evident [25].

Linear correlations have been observed between left ventricular (LV) mass, left ventricular posterior wall dimensions (LVPWDs), and LV mass index (normalized by body surface area) in both male and female patients as the severity of HCM increases (Supplementary Figure S1A). In contrast, a correlation between left ventricular internal diameter (LVID), left ventricular internal diameter at systole (LVID), and LVPWDs has only been observed in female patients with FD, not in males (Supplementary Figure S1B).

Next, we investigated the correlation between the age at which patients with FD were diagnosed with HCM and the age at which FD-specific therapy (ERT or ST) was initiated (Figure 1B,C). In this cohort, it is common for patients with FD to initiate ERT after the onset of hypertrophic cardiomyopathy. This pattern is consistent among both male and female patients. Consequently, the early diagnosis of FD, particularly before the onset of symptoms, remains a significant challenge in the effective management of the disease.

### 3.2. Principal Component Analysis (PCA) Reveals a Distinct Separation of Inflammatory Biomarkers and Growth Factors Between Male and Female Patients with FD

**Figure 2:**
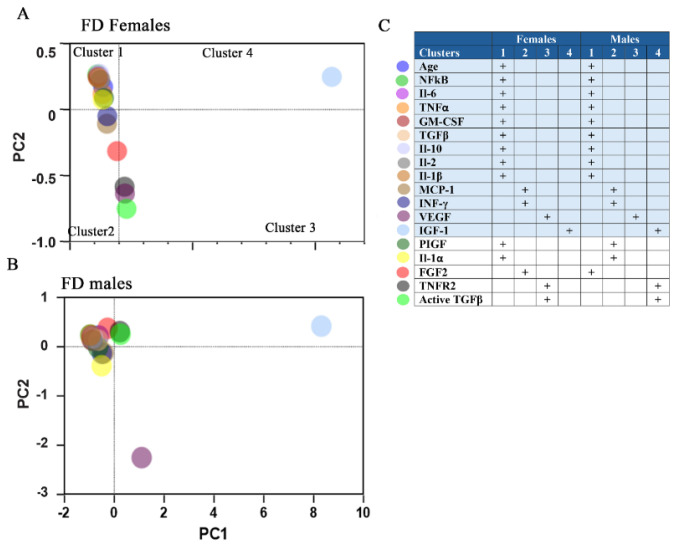
Multivariate statistical analysis. Principle component analysis (PCA) was used to create a two-dimensional cluster plot of PC-1 and PC-2 for 18 biomarkers. The plot shows the PC1 vs. PC2 score scatter plot for female (**A**) and male (**B**) patients with FD, with each dot representing a different biomarker. The table represents biomarkers combined in Cluster 1, where PC1 is negative, and PC2 is positive. (**C**)**.** The list of biomarkers represents the categorization of inflammatory biomarkers into clusters using PCA. “Blue background” indicates the biomarkers with similar distribution in female and male patients with FD, while “white” refers to biomarkers in distinct clusters in the female and male groups.

Multivariate PCA is a widely used statistical method designed to differentiate the variance within a data set in which individual points (samples) with similar variation characteristics can be clustered together, and the differences between sample groups can be visualized. PCA was used to identify underlying dimensions of inflammatory and growth factor activity from 18 biomarkers measured in 45 FD patients and included the following parameters: age, FGF2, NF-kB, Il-6, TNF-α, TNFR2, MCP-1 (CCL2), INF-γ, VEGF, GM-CSF, PIGF, TGF-β, active TGF-β, IGF-1, IL-10, Il-2, IL-1α, and IL-1β. Figure 2 represents two 2D plots of the PCA results with two principal components, PC1 vs. PC2, that differentiated inflammatory biomarkers and growth factors between female and male cohorts (Figure 2A,B). The clustering reflects the presence of one distinct cluster (cluster 1) with biomarkers that overlap for both groups (age, NF-kB, Il-6, TNF-α, GM-CSF, TGFβ, Il-10, Il-2, Il-1β). Additionally, cluster 1 in the female group also included PIGF and IL-1α. FGF2 is specific for the male cluster 1 group (Figure 2). A distinct subgroup characterized by unique biomarker patterns includes MCP-1, INF-γ, IL-1α, TNRF2, and active TGF-β (Figure 2). Cluster 2 represents MCP-1 and INF-γ for both groups, but FGF2 only for female cluster 2. TNRF2 and active TGF-β are present in female cluster 3 and in male cluster 4.

### 3.3. Il-2, Il-10, TNF-α, and IFN-γ Are Elevated in Both Female and Male Patients with FD

**Figure 3:**
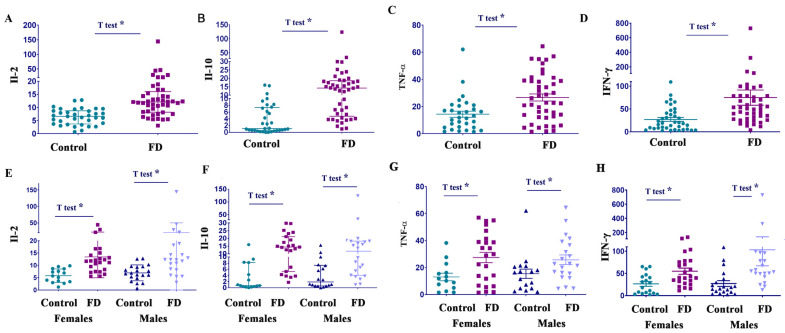
Circulated IL-2, IL-10, TNF-α, and IFN-γ levels are elevated in FD patients. (**A**) Interleukin 2 (IL-2) levels, control vs. FD. Statistical analysis using an unpaired *t*-test demonstrated a significant difference between control and FD cohorts. * *p* < 0.05. (**B**) Interleukin 10 (IL-10) levels, control vs. FD. Statistical analysis using unpaired *t*-test and F-test to compare variance demonstrated a significant difference between control and FD cohorts. * *p* < 0.01. (**C**) Tumor necrosis factor (TNF-α) levels, control vs. FD. The unpaired *t*-test demonstrated a significant difference between healthy control and FD cohorts. * *p* = 0.001. (**D**) Interferon (IFN-γ) levels, control vs. FD. The unpaired *t*-test, * *p* < 0.05. (**E**) Comparing the Il-2 levels in control females and female patients with FD, control males and male patients with FD. * *p* < 0.05 *t*-test control females vs. FD females and control males vs. FD males. (**F**) Il-10 levels in control females vs. female patients, and control males vs. male patients with FD. * *t*-test *p* < 0.01. (**G**) Comparing TNF-α levels in control females vs. female patients, and control males vs. male patients with FD. * *t*-test *p* < 0.01. (**H**) IFN-γ levels in control females and female patients with FD, control males and male patients with FD. * *p* < 0.05 *t*-test.

The analysis of biomarkers from cluster 1 showed that Il-2 and IL-10 were significantly higher in the FD cohort than in the healthy controls (Figure 3A–C, Table 1). The marker of proinflammatory activity, granulocyte-macrophage colony-stimulating factor (GM-CSF), was found to be markedly elevated in 5 out of 45 patients (1 female and 4 males). Furthermore, with the wide range of GM-CSF levels in healthy controls, the F-test, not the *t*-test, confirmed statistically significant differences in “means” (Supplementary Figure S1).

The role of TNF-α as a mediator of inflammatory and immune functions is still debatable in FD. Some studies have shown increased TNF-α levels, while others have found no significant difference between the TNF-α levels of FD patients and healthy individuals [28,29]. In this study, analysis of plasma from healthy control and FD cohorts demonstrated that the TNF-α level was significantly elevated in patients with FD (Figure 3C and Table 1). In cluster 1, inflammatory biomarkers such as NF-kB and IL-6 were not elevated compared to the healthy controls (Table 1).

Analysis of Cluster 2 revealed that levels of the proinflammatory cytokine IFN-γ were significantly higher in the FD cohort compared to the healthy controls. Further analysis showed that IL-2, Il-10, TNF-α, and IFN-γ increased significantly in female and male patients with FD compared with healthy controls (Figure 3E–H, and Table 1).

### 3.4. IL-1α Levels Are Elevated in Female Patients with FD

**Figure 4:**
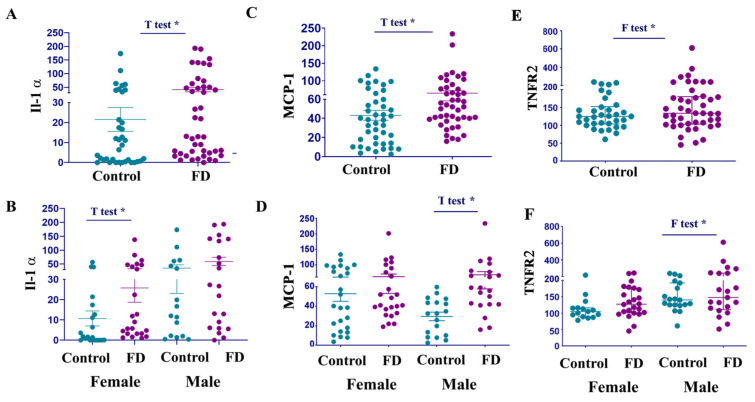
Circulated Il-1α, MCP-1, and TNRF1 levels are elevated in patients with FD. (**A**) Interleukin 1α (Il-1 α) levels, control vs. FD. (**B**) Comparing the Il-1 α levels in female controls vs. FD, male controls vs. male patients with FD. * *p* < 0.05. (**C**) Monocyte chemoattractant protein 1 (MCP1) levels, control vs. FD. *t*-test; * *p* < 0.05. (**D**) Comparing the MCP-1 in female controls vs. FD, male controls vs. FD. * *p* < 0.05. (**E**) TNFR2, control vs. FD. F test, * *p* < 0.05. (**F**) Tumor necrosis factor α receptor (TNFR2) in female controls vs. FD, and comparing male controls vs. FD. F-test verified a significant difference between control males vs. FD males * *p* < 0.05.

Of the 11 members of the IL-1 family, the two most prominent members are proinflammatory cytokine IL-1α and IL-1β, related to pyroptosis and programming cell death pathways [30,31,32]. Moreover, IL-1α, with diverse physiological functions, also plays a significant role in myocardial inflammation related to disrupting calcium and β-adrenergic receptor signaling, mitochondrial function, and increasing nitric oxide production [30]. Like TNF-α, IL-1α was significantly higher in FD females but not in the FD male groups (Figure 4C,D, Table 1). We did not observe a significant increase in IL-1β compared to healthy controls. However, it is important to note that due to the limited sensitivity of the IL-1β ELISA test, some healthy controls and FD patients had undetectable levels of IL-1β. Moreover, IL-1β is released through different mechanisms, depending on the intensity of the proinflammatory stimuli. For example, IL-1β reaches a peak during the early stage of cytokine storms; however, 12–24 h later, the level of cytokine in the bloodstream rapidly decreases [33,34].

### 3.5. MCP-1 and TNFR2 Are Elevated in Males with FD

Elevated MCP-1 levels in the blood are linked to an increased risk of cardiovascular diseases in the general population and play a crucial role in conditions such as myocarditis, ischemia/reperfusion injury, and transplant rejection [35]. Furthermore, MCP-1 is a potential biomarker for evaluating cardio manifestations in patients with FD [36]. Our findings corroborate previous research, showing a significant increase in MCP-1 levels in FD patients compared to healthy controls. Notably, the sex-stratified analysis revealed that elevated MCP-1 levels are more prevalent in males than females (Figure 4E and Table 1). Tumor necrosis factor α receptor (TNFR2) is a biomarker of classical proinflammatory activity. Previously, it has been reported that elevated TNFR2 correlates with myocardial fibrosis and renal disease in patients with FD [37]. In our cohort, TNFR2 was elevated in several patients; therefore, the F-test, not the *t*-test, showed significant differences in mean values (Figure 4E,F, Table 1).

### 3.6. Growth Factors FGF2 and IGF-1, but Not PIGF, Are Elevated in FD

**Figure 5:**
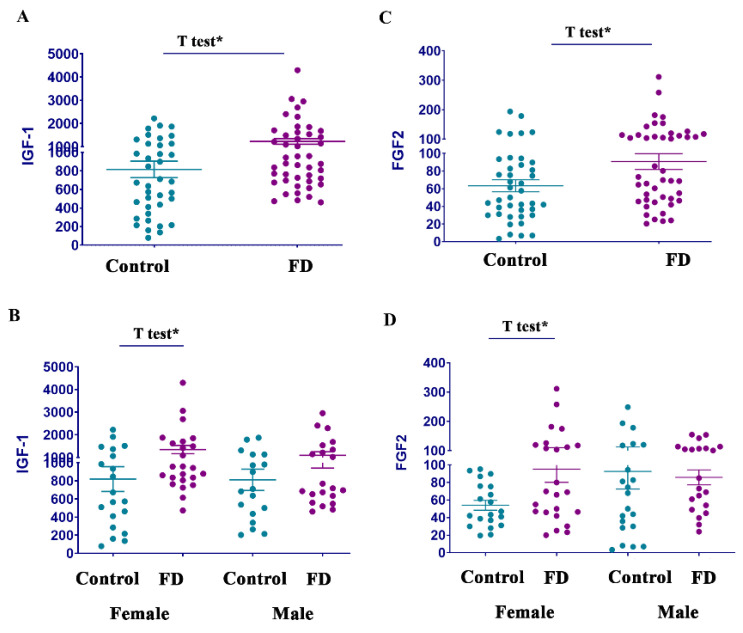
Circulated levels of FGF2 and IGF-1 are elevated in patients with FD. (**A**) Insulin-like growth factor 1 (IGF-1) levels control vs. FD. Statistical analysis using an unpaired *t*-test demonstrated a significant difference between control and FD cohorts. * *p* < 0.05 *t*-test. (**B**) IGF-1 levels in control females and female patients with FD, as well as in control males and male patients with FD. * *p* < 0.05, *t*-test comparison between control and FD females. (**C**) Fibroblast growth factor 2 (FGF2) levels control vs. FD. Statistical analysis using an unpaired *t*-test to compare cohorts demonstrated a significantly increased level in FD. * *p* < 0.05 (**D**) FGF2 level in control females and female patients with FD, control males and male patients with FD. * *p* < 0.05, *t*-test comparison between control and FD females.

Growth factors, including IGF-1, FGF2, and PIGF, contribute to cardiac development, diseases, and repairs and play an essential role in the initiation and promotion of cardiomyocyte and fibroblast differentiation [38]. Moreover, cardio-hypertrophic remodeling FGF2 and IGF-1 biomarkers are associated with inflammatory activity. Analysis of plasma revealed significantly higher levels of FGF2 and IGF-1 in patients with FD (Figure 5 and Table 1). Further analysis showed that FGF2 and IGF-1 were more noticeably elevated in FD females. Circulated PIGF levels in female and male patients with FD were no different than in healthy controls (Table 1).

### 3.7. Associations of Inflammatory Biomarkers and Growth Factors with HCM in FD

To investigate whether any of these biomarkers are associated with cardiomyopathy, we further adjusted FD cohorts: patients without and with cardiomyopathy: HCM(−) and HCM(+), respectively. Among 11 inflammatory biomarkers, a high level of MCP1 was significantly associated with HCM in FD females. By comparison, the elevation of Il-6 and TNF-α showed a significant association with HCM in FD males (Table 2). When stratifying by gender with or without HCM, a decrease in sample size led to the use of the F-test to compare two variances. Thus, GM-CSF and IL-1β were the biomarkers with a suggestive association with HCM in both male and female patients with FD. Elevations of Il-2, Il-10, and IFN-γ were associated with HCM in FD males (Table 2). The levels of MCP-1 were found to be higher in males with HCM, but the difference was not statistically significant.

### 3.8. Correlation of Lyso-Gb-3 and Inflammatory Biomarkers or Growth Factors in FD Patients with Cardiac Involvement

**Figure 6:**
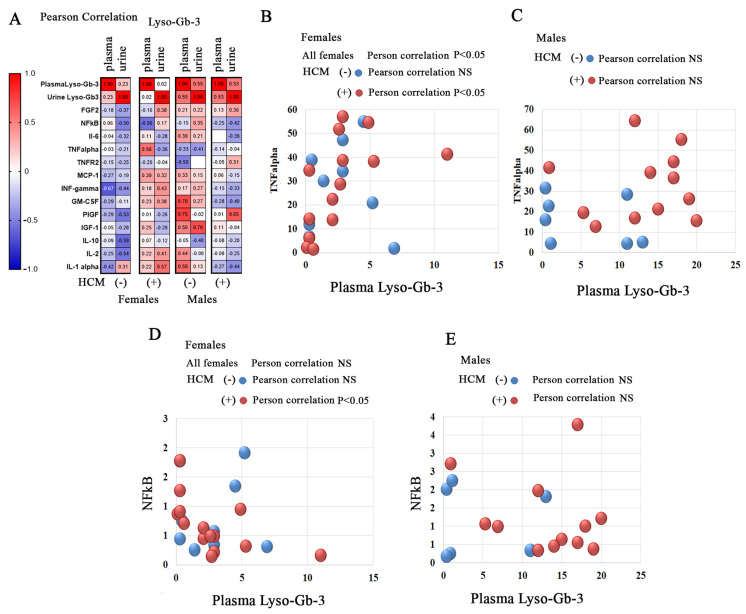
(**A**) Pearson correlation coefficient matrix presentation for 13 plasma biomarkers with plasma lyso-Gb-3 and urine levels of Gb-3 in cohorts: FD females without HCM(−) and with HCM(+), FD males with the absence of HCM(−), and patients with HCM(+). (**B**,**C**) Scatterplot analysis of correlation of TNFα and plasma lyso-Gb-3 in females (**B**) and males (**C**) with and without HCM. (**D**,**E**) Scatterplot analysis of correlation of NF-kB and plasma lyso-Gb-3 in females (**D**) and in FD male patients with and without HCM (**E**).

Gb-3 and lyso-Gb-3 have cytotoxic, proinflammatory, and profibrotic effects on FD pathology [12,39,40]. The deposition of lyso-Gb-3 within the myocardium has a negative impact on cardiac function and is associated with cardiovascular diseases [9]. A correlation matrix analysis has been used to study the relationship between lyso-Gb-3 and inflammatory biomarkers, or growth factors in FD patients. The analysis revealed diverse patterns in the Pearson linear correlation between lyso-Gb3 and inflammatory biomarkers in FD female and male patients with and without HCM (Figure 6A and Supplementary Table S2). A significant positive linear correlation was observed between lyso-Gb-3 and TNF-α in FD females (Figure 6). When the stratification of FD cohorts was carried out based on the presence of HCM, a positive linear correlation was noted between plasma lyso-Gb-3 and TNF-α among FD females with HCM. Additionally, the circulated level of NF-kB was found to correlate with plasma lyso-Gb-3 only in female patients HCM(+) (Figure 6A,D and Supplementary Table S2). A significant correlation has been found between PIGF and urine Gb-3 in male patients with FD and HCM (Supplementary Table S2).

### 3.9. Sex-Related Association Between HCM and the NF-kB-INFγ Pathways, and TNFα-TNFR2 Signaling Cluster

**Figure 7:**
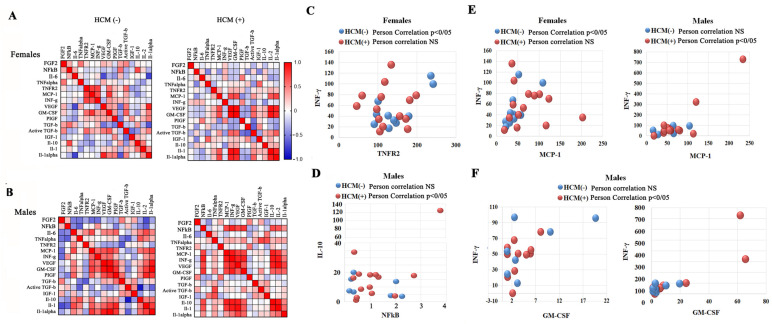
Correlation matrix and hierarchical clustering. (**A**,**B**) Correlation coefficients for measurements of biomarkers are visualized by tile-color intensities (red color, strong; deep blue color, negative correlation). FD females without cardiomyopathy HCM(−) and with HCM(+). A correlation coefficient ≥ 0.8 indicates strong positive relationships; a correlation coefficient = between 0.5 and 0.7 indicates a moderate positive relationship; a correlation coefficient less than 0.5 indicates variables with a low correlation. Correlation is accepted as significant differences by Pearson’s correlation *p*-values < 0.05. (**C**) A scatterplot analysis of the correlation of INF-γ and TNFR2 in FD female patients without and with HCM. (**D**) A scatterplot analysis of the correlation of IL-10 and NF-kB in FD male patients without and with HCM. (**E**) A scatterplot analysis examined the correlation between INF-γ and MCP-1 in females and male patients with and without HCM. (**F**) A scatterplot analysis examined the correlation between INF-γ and GM-CSF in male patients with and without HCM. On the left side, the scatterplot displays GM-CSF on the X-axis, with a maximum value of 22 pg/mL, and INF-γ on the Y-axis, with a maximum value of 110 pg/mL. On the right side, the scatterplot is displayed without the limitation of the X- and Y-axis.

Next, we investigated the data set of inflammatory biomarkers and growth factors by calculating the Pearson correlations between biomarkers from female and male patients with and without HCM (Figure 7A). Figure 7 illustrates the heat map of correlation matrix analysis between 17 biomarkers. Significant differences in the results of Pearson correlation analysis have been found between biomarkers in males and females with and without HCM. Thus, a significant positive correlation was found between INF-γ and TNFR2 levels in FD female patients without HCM. However, in females with HCM, INF-γ levels were generally elevated, regardless of low TNFR2 levels (Figure 7C). In male patients with FD, there was no correlation between INF-γ and TNFR2 with or without HCM. However, in male patients with FD, significant positive correlations were observed in several cluster groups, including a prominent correlation between INF-γ and MCP-1, Il-10 and NF-kB, and INF-γ and GM-CSF (Figure 7D–F).

**Figure 8:**
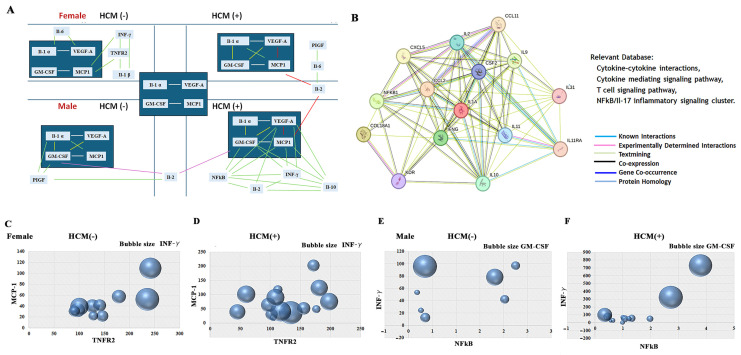
Signaling clusters and protein–protein interaction map staged the sex-related association with cardiomyopathy (HCM) in Fabry disease. (**A**) Multi-level networks in FD cardiomyopathy based on Pearson correlation analysis. (**B**) Protein–protein interaction networks suggest cytokine activation pathways play an essential role in HCM in male patients with FD. Protein–protein interaction network between NF-kB, Il-2, Il-20, INF-γ (IFNG), MCP-1 (CCL2), GM-CSF (CSF2), VEGF (KDR), and Il-1 α (IL1A) has been developed using STRING. (**C**,**D**) A bubble chart has been created using the scatter 3D plots in Excel and represents the correlation between TNFR2 (X-axis), MCP-1 (Y-axis), and INF-γ (bubble size represents the concentration of the biomarker) in female patients without (HCM−) and with HCM (HCM+). (**E**,**F**) The bubble scatter 3D plots represent the correlation between NF-kB (X-axis), INF-γ (Y-axis), and GM-CSF (bubble presentation) in male patients without (HCM−) and with HCM (HCM+).

Multi-level networks based on Pearson correlation data confirm the significant positive linear correlations between GM-CSF and MCP1, Il-1α, and VEGF in all FD groups, independent of gender or HCM status (Figure 8A, Supplementary Table S3). Linear correlation between GM-CSF and Il-1α, and GM-CSF and VEGF biomarkers was detected in FD females with HCM and FD males regardless of HCM status (Figure 8A, Supplementary Table S3). A significant linear correlation was observed between MCP-1 and VEGF, as well as between MCP-1 and IL-2, in both male and female patients with HCM. This indicates that the relationship between MCP-1, VEGF, and IL-2 is related to cardiomyopathy, not FD (Figure 8A and Supplementary Table S3).

The most significant spike of linear correlations between inflammatory biomarkers was detected in male patients with FD and HCM, particularly actively involving the inflammatory cluster of GM-CSF, MCP-1, as well as the growth factor VEGF-A (Figure 8A, Supplementary Table S3). Moreover, in the presence of HCM pathology, the network web advances with the emergence of new inflammatory players: NF-kB, IL-2, IL-10, and INF-γ (Figure 8A). The “STRING” enrichment analysis of inflammatory biomarkers which are activated in male FD patients with HCM confirms the involvement of cytokine–cytokine interaction, the cytokine-mediated signaling pathway, the T-cell signaling pathway, and the NF-kB/Il-17 signaling pathway in patients with FD with the presence of HCM.

Cluster correlation between MCP-1, TNFR2, and INFγ was only found in FD females without HCM, verifying that patients with elevated levels of MCP-1 also have elevated levels of TNFR2 and INFγ. No correlation was detected among MCP-1, TNFR2, and INFγ biomarkers in patients with HCM, as illustrated in the 3D bubble plot (Figure 8C,D). In male patients with FD, the most significant cluster correlation was detected among three biomarkers, NF-kB, INFγ, and GM-CSF, which highlighted the essential role of activation of inflammation in HCM progression (Figure 8E,F).

## 4. Discussion

Cardiovascular complications are most frequently encountered in FD, not only contributing substantially to morbidity but also being the leading cause of premature death in male and female patients with FD [41]. The manifestations of cardiac involvement are left ventricular hypertrophy (LVH), diastolic dysfunction, microvascular angina, and HCM [42,43]. In this study, we aimed to identify blood-circulated biomarkers for the early diagnosis and prediction of HCM in patients with FD. The study’s secondary endpoints addressed the role of sex differences in the activation of inflammation and growth factors and their correlation with cardiac pathology in FD patients. The cardiac involvement was assessed using standard clinical imaging tools, EKG, echocardiogram, and cardiac MRI with late gadolinium enhancement (LGE-CMR), to evaluate HCM and myocardial fibrosis [44,45,46]. Cardiac involvement was stratified using LVPWd, LV mass, and LV mass/BSA adapted from Olivotto et al.’s study and was described previously [12,47]. Although LV mass is greater in men than in women due to a correlation with body size, adjustments between LVmass, LVPWD, and LV/BSA correlated with the severity of HCM in both male and female patients with FD, as we expected.

Females with FD were described as largely asymptomatic until 2005, when an updated version of “Harrison’s Principles of Internal Medicine” acknowledged that up to 70% of heterozygous females may exhibit clinical manifestations, including cardiac disease [48]. The common belief that FD symptoms in males appear during childhood or teenage years and symptoms appear ten years later in females is not always accurate [49,50]. Thus, HCM progression in females with FD might differ substantially [23,51]. In females, the progression of the disease may be undetectable for a prolonged period, leading to delayed recognition of the heart disease. In our cohort, two female patients in their early 20s were diagnosed with HCM, and seven females started enzyme replacement therapy (ERT) before the age of 40 due to heart complications. Because diagnostic delays can result in significant disease progression, initial cardiac evaluation for female patients with FD should include not only the assessments for left ventricular hypertrophy but also the extent of fibrosis [23].

The progression of HCM and cardiac fibrosis involves inflammation characterized by immune cell infiltration into myocardial tissue with the secretion of cytokines. This cytokine storm promotes further cardiac inflammation, promoting the prolonged process of LV progressive dysfunction [47], and myocardial fibrosis [52]. Proinflammatory cytokines such as IL-1α, IL-1β, TNF-α, and TNFR2 are elevated in animal models and patients with chronic heart failure [15]. TNF-α, secreted by cardiac myocytes and macrophages, induces autocrine and paracrine activation and may perpetuate inflammation. This process triggers a series of proinflammatory signals, which, in the context of myocardial tissue, can unleash apoptosis/cell death pathways in myocytes and endothelial cells [15]. A significant increase in circulated TNF-α has previously been identified in patients with FD, proposing that TNF-α is a potential biomarker for monitoring cardiac outcomes in patients [29,36,53]. Our findings support previous research showing that elevated levels of TNF-α were present in both male and female FD patients compared to healthy controls. However, in males, the increase in TNF-α significantly correlates with the presence of HCM, whereas this correlation is less evident in females. Notably, a positive linear correlation between TNF-α and plasma lyso-Gb-3 levels has been confirmed in females.

The TNFα cytoplasmic membrane receptors (TNFR1 and TNFR2) regulate the TNFα-TNFR signaling cascade with opposing effects in cardio-inflammatory conditions. TNFα/TNFR1 is associated with proinflammatory effects and cell death, while TNFα/TNFR2 is linked to cardioprotective processes. Previously, it has been suggested that patients with FD and LVH also had higher plasma levels of TNFR2, while patients with late gadolinium enhancement demonstrated increased TNFR1 and TNFR2 levels [54].

We observed elevated TNFR2 levels in FD patients, with gender influencing the circulating levels of TNFR2. FD females who do not have HCM showed significantly higher levels of circulating TNFR2 and demonstrated a positive correlation between TNFR2 with MCP-1 and INF-γ. In contrast, FD females without HCM showed a lack of correlation between MCP-1, INF-γ, and TNFR2, although MCP-1 was significantly higher. Given the study’s limited sample size, our hypothesis—drawn from the existing literature—suggests that TNFα and TNFR2 receptor interactions might activate a protective mechanism regulating the cardiac proinflammatory response through NF-κB and PI3K/Akt pathways, leading to the expression of MCP-1 which recruits monocytes and macrophages to inflammation sites [55,56].

Patients with HCM experience a “chronic low-grade” inflammatory state with elevated levels of NF-kB that promote the expression of TNF-α, as well as interleukins such as IL-1β, IL-1RA, IL-6, IL-10, and MCP-1 [57,58]. NF-kB upregulation in the myocardium initiates the invasion of inflammatory cells into the myocardium, the production of proinflammatory cytokines, and fibroblast differentiation to form fibrosis tissue [59]. The circulating level of NF-kB was not elevated in FD compared to healthy controls; however, interleukins upregulated by NF-kB, such as IL-1β, TNF-α, IL-6, and MCP-1, were elevated in FD patients with HCM.

Our findings confirm prior research showing that GM-CSF, VEGF-A, and MCP-1 levels are elevated in FD patients, indicating an inflammatory state. We noted increased IL-1α in males and a significant increase in females with FD, underscoring the pervasive inflammation in FD. Both genders, irrespective of HCM status, showed a linear correlation between circulating IL-1α and both VEGF-A and GM-CSF, further validating the inflammatory nature of FD. Detailed cytokine profiling demonstrated complex interactions contributing to cardiomyopathy in FD. Chronic inflammation plays a significant role in the progression of FD, affecting not only cardiovascular health but also kidney function and cerebrovascular complications [37,60]. An analysis of signaling clusters and the mapping of cytokine interactions revealed that the complexity of these interactions contributes to cardiomyopathy in patients with FD. In female patients with HCM, the cytokine interaction cluster includes IL-1α, VEGF-A, GM-CSF, and MCP-1. In contrast, male patients with HCM show significantly larger cytokine interaction clusters, which include key players of inflammatory pathways such as NF-kB, INF-γ, IL-2, and IL-10. Four key pathways were identified as highly prominent in HCM in FD, cytokine–cytokine interactions, cytokine-mediating pathways, T-cell signaling pathways, and the NF-kB inflammatory signaling cluster, according to the protein interaction network.

Recent proteomic studies have demonstrated the differential expression of proteins related to endothelial dysfunction and cerebrovascular complications of FD that respond to maintaining endothelial integrity (CRACD, HRG65), heme-hemoglobin metabolism, oxidative stress (TTHY, QSOX1, HEMO, CATA), and complement regulation (IGHG3, CO3, FCN2, FHR1) [53,61]. Recent exploratory studies of microRNAs (miRNAs) have indicated that circulating miRNAs are associated with stress-induced apoptosis, abnormal vascularization, and inflammation and play a significant role in the progression of disease in FD [62,63]. For example, miR-184 targeting HIF1AN, Rab1a, and Rab31 may reflect cardiac or renal pathology in FD [64,65,66]. Additionally, miR-181a-2-3p, which targets Parkin, and miR-30d-5p, which suppresses Beclin 1, are linked to the alteration of autophagy in FD [67]. miR-361, miR-146, and miR-126, which target VEGF, Nox4, and p85β, respectively, may disrupt vascular development and lead to abnormal vascularization in FD [62].

While our study primarily focused on inflammatory biomarkers, we also studied several growth factors associated with the differentiation of cardiomyocytes. Growth factors FGF2 and IGF-1 were elevated in female patients; however, no correlation was observed with HCM or Lyso-Gb-3 levels. Elevated levels of angiogenesis-related proteins such as FGF2, VEGF-A, and VEGF-C have been described in patients with FD, with no correlation to Lyso-Gb-3 [68,69]. Among these proteins, VEGF-A is the most significant factor associated with HCM pathology in both male and female patients [12].

The deposition of Gb-3 and Lyso-Gb-3 within the myocardium, in the cardiac valves, cardiomyocytes, nerves, and coronary arteries, affects cardiac function and accompanying cardiovascular pathology [7,9]. At the cellular level, the accumulation of Gb-3 and Lyso-Gb-3 impairs autophagy–lysosomal function and mitochondrial function, which also leads to chronic inflammation, cell death, and cardiac tissue and to the initiation of the differentiation of cardiac fibroblasts and end-stage fibrosis [67,70,71]. Similarly to growth factors, there was no correlation between Lyso-Gb-3 and inflammatory biomarkers, with the exception of a correlation observed with NF-kB and TNFα in female patients with HCM. However, it is important to note that in this study, only two patients were naïve: one without HCM and one with HCM. All other patients were receiving ERT or chaperone therapy, which resulted in decreased levels of Gb-3 and Lyso-Gb-3.

Males tend to experience a more pronounced inflammatory response compared to females, primarily due to hormonal factors, particularly due to testosterone, which can lead to significant cardiac damage and fibrosis in men with cardiomyopathy [72,73]. In contrast, females often demonstrate a more robust regulatory immune response, which can help mitigate inflammation more effectively than in males [20,74]. Another factor contributing to sex-related differences in disease progression is the X-linked nature of the disease, leading to a complex interplay of inflammation. In FD, hemizygous males generally experience more severe symptoms and a more predictable risk of disease pathology, including HCM. Conversely, heterozygous females present with a wide range of symptoms due to the inactivation of either mutated or unmutated *GLA* on the X chromosome. Research indicates that FD females who repress the non-mutant allele tend to show a higher severity score for the disease [4]. However, the roles of X-inactivation, GLA variants, and DNA methylation in influencing inflammatory responses related to cardiac pathology in FD have yet to be fully explored [4,51,75,76]. Therefore, it is important to understand how *GLA* pathogenic variants and DNA methylation upregulate inflammatory genes within the context of cardiomyopathy. Additionally, the implications of X-inactivation patterns in heterozygous females could significantly impact disease severity in FD.

Glycosphingolipids (GSLs), including Gb-3 and lyso-Gb-3, have been implicated as key mediators of inflammation in cardiovascular pathology, leading to oxidative stress and macrophage activation with cytokine release [77]. The inflammatory response due to GSL accumulation is crucial in the development of cardiovascular dysfunction, emphasizing the necessity for therapeutic strategies that target inflammatory pathways. It is necessary to note that not only the accumulation of Gb3 and lyso-Gb3 in multiple organs but also circulating GSL contributes to the inflammatory response, which further drives myocardial fibrosis, hypertrophy, and eventual heart failure [78].

Given the inflammatory nature of Fabry cardiomyopathy, anti-inflammatory therapies may complement FD-targeted therapies, such as enzyme replacement therapy (ERT) and chaperone therapies, to improve cardiovascular outcomes. Targeting inflammation in cardiovascular diseases has emerged as a promising therapeutic strategy. Colchicine, an anti-inflammatory drug traditionally used to treat gout and pericarditis, has shown efficacy in reducing cardiovascular risk by inhibiting microtubule polymerization and suppressing cytokine release, including IL-1β and IL-6 [79]. The Colchicine Cardiovascular Outcomes Trial (COLCOT) and Low-Dose Colchicine for Secondary Prevention of Cardiovascular Disease (LoDoCo2) trial demonstrated that low-dose colchicine significantly reduces the risk of major adverse cardiovascular events, including myocardial infarction and stroke, by mitigating vascular inflammation [79,80]. Additionally, IL-1β inhibitors such as canakinumab have been shown to reduce cardiovascular events in patients with elevated inflammatory markers [81]. These findings support the role of inflammation as a therapeutic target in cardiovascular diseases, including Fabry cardiomyopathy, where adjunctive anti-inflammatory therapies may improve clinical outcomes when combined with disease-specific treatments.

## 5. Conclusions

Distinct variations in inflammatory responses between male and female patients with FD are evident. In males, increased cytokine levels and NFκB signaling activation suggest involvement in the inflammatory processes contributing to HCM remodeling. In contrast, females with HCM display a distinct clustering of TNFα, TNFR2, and TGFβ signaling pathways, potentially elucidating the precocious development of fibrosis in these patients. The pivotal roles these cytokines and growth factors play may render them valuable as biomarkers for the early detection and clinical staging of HCM. Such insights are crucial for healthcare providers to develop strategies for the effective management of FD patients with cardiac manifestations.

## Figures and Tables

**Table 1:** Immunological biomarkers and growth factors among healthy controls vs. female or male patients with FD. The statistical analysis presents mean ± SEM, and “No” means there is no statistically significant difference. * Student’s *t*-test or ^#^ F-test analysis.

	CNT vs. FD Females			CNT vs. FD Males		
	CNT	FD	Statistic *	CNT	FD	Statistic *
Il-2	5.8 ± 0.7	13.41 ± 1.1	* *p* < 0.0005	6.9 ± 0.7	20.24 ± 6.2	* *p* < 0.05
Il-10	3.5 ± 1.2	14.3 ± 1.7	* *p* < 0.0001	4.1 ± 1.1	16.1 ± 5.3	* *p* < 0.05
IFN-γ	26.4 ± 5.4	55.0 ± 6.91	* *p* < 0.001	27.3 ± 6.5	103 ± 36	* *p* < 0.05
TNF-α	13.0 ± 2.8	27.3 ± 3.8	* *p* < 0.01	15.0 ± 3.4	25.9 ± 3.5	* *p* < 0.05
IL-1α	10.7 ± 3.6	25.8 ± 7.1	* *p* < 0.05	35.1 ± 12	59.4 ± 14.9	No
MCP-1	52.8 ± 7.7	61.9 ± 8.7	*p* = 0.22	29.5 ± 4.3	68.05 ± 10.2	* *p* = 0.001
GM-CSF	1.9 ± 0.2	2.5 ± 0.8	^#^ *p* < 0.0001	5.3 ± 1.6	11.0 ± 4.1	^#^ *p* < 0.0001
TNFR2	113 ± 9	135.4 ± 10	* *p* = 0.06	149.8 ± 10	191.8 ± 28	^#^ *p* < 0.0001
Il-6	10.4 ± 1.5	7.8 ± 0.9	*p* = 0.07	9.4 ± 1.3	11.4 ± 1.3	*p* = 0.17
NF-kB	0.74 ± 0.1	0.76 ± 0.1	*p* = 0.49	1.04 ± 0.2	1.14 ± 0.2	*p* = 0.18
IL-1β	4.3 ± 1.2	4.2 ± 0.9	*p* = 0.46	3.6 ± 0.9	3.06 ± 1.2	*p* = 0.45
IGF-1	819 ± 136	1350 ± 183	* *p* < 0.05	811 ± 117	1096 ± 155	*p* = 0.08
FGF2	54.1 ± 5.5	95.2 ± 15.1	* *p* < 0.05	78.9 ± 14.5	85.8 ± 8.5	*p* = 0.32
PIGF	35.0 ± 4.6	30.7 ± 3.5	*p* = 0.20	32.7 ± 6.1	38.3 ± 4.1	*p* = 0.22

**Table 2:** Levels of immunological biomarkers and growth factors among female and male patients with FD without cardiomyopathy (HCM−) and with cardiomyopathy (HCM+). The statistical analysis presents mean ± SEM, and “No” means there is no statistically significant difference. * Student’s *t*-test or ^#^ F-test analysis.

	Females			Males		
	HCM(−)	HCM (+)	Statistic *	HCM(−)	HCM (+)	Statistic *
Il-2	14.93+/−2.02	12.32+/−1.02	*p* = 0.23	13.5+/−2.92	23.6+/−9.7	^#^ *p* = 0.001
Il-10	13.7+/−2.9	13.9+/−2.3	*p* = 0.48	10.1+/−2.4	21.1+/−8.3	^#^ *p* < 0.001
IFN-γ	50.9+/−10.4	57.9+/−9.4	*p* = 0.31	57.8+/−12.7	128+/−54.5	^#^ *p* < 0.001
TNF-α	26.4+/−5.8	29.0+/−5.7	*p* = 0.32	16.4+/−6.7	30.1+/−5.2	*p* < 0.05
IL-1α	23.0+/−8.7	27.9+/−10.8	*p* = 0.36	36.7+/−18.4	70.7+/−20	*p* = 0.14
MCP-1	44.7+/−8.06	74.2+/−13.2	* *p* < 0.05	54.4+/−11.1	74.8+/−14.3	*p* = 0.18
GM-CSF	1.5+/−0.3	3.3+/−1.4	^#^ *p* < 0.001	5.75+/−2.6	13.6+/−5.8	^#^ *p* < 0.001
TNFR2	149 +/−17.2	126+/−12.2	*p* = 0.14	139+/−31	218+/−37.8	*p* = 0.09
Il-6	6.8+/−1.1	8.5+/−1.1	*p* = 0.2	7.2+/−0.6	13.6+/−1.7	*p < 0.05
NF-kB	0.8+/−0.2	0.67+/−0.1	*p* = 0.24	1.04+/−0.3	1.2+/−0.3	*p* = 0.36
IL-1β	2.9+/−1.0	5.4+/−1.94	^#^ *p* < 0.05	3.9+/−3.1	2.6+/−0.9	^#^ *p* < 0.05

## Data Availability

The data presented in this study are available on request from the corresponding author.

## Supplementary Materials

The following supporting information can be downloaded at https://www.mdpi.com/article/10.3390/cells14050322/s1: Figure S1: (A) Multivariable analysis between Left ventricular mass (LV mass), Left ventricular posterior wall end diastole and end systole LVPWd, and LV mass/BSA between female and male FD patients with and without HCM. (B) Multivariable analysis between left ventricular internal dimension (LVID), end-systolic (LVIDs), and LVPWd in female and male FD patients with and without HCM. (C) Left: GM-CSF levels, control vs. FD. Statistical analysis using unpaired T-test and F-test to compare variance. F-test demonstrated a significant difference between control and FD cohorts. * *p* < 0.05. Right GM-CSF levels, control vs. FD stratify by sex. F-test * *p* < 0.01; Table S1: Demographics, genotypes, and diagnostic characteristics of patients with Fabry diseases. “#” patients were categorized as a “borderline” due to abnormal EKG. “&” Patients with the severe form of HCM; Table S2: Correlation matrix analysis to compare inflammatory biomarkers and growth factors with plasma or urine Lyso-Gb-3 levels in female and male patients with FD. Additionally, the data was divided into cohorts: “no cardio pathology” HCM(−) and patients with cardiomyopathy HCM(+). The values in the table are for a two-tailed Pearson correlation test with r and *p* values. Where the r values indicated negative linear correlation (−1 to −0.5), positive linear correlation (0.5 to 1), or absence of linear correlation (between −0.5 to 0.5). *p* value significant differences *p* < 0.05, Pearson correlation, two tails highlighted with color; Table S3: The Pearson correlation analysis matrix assesses the relationship between two variables in a data set. The table displays the *p*-value for the two tails Pearson correlation. *p* < 0.05 significant linear correlation.

## Abbreviations

The following abbreviations are used in this manuscript:

IL-2	Interleukin-2
IL-10	Interleukin-10
IFN-γ	Interferon gamma
TNF-α	Tumor necrosis factor-alpha
IL-1α	Interleukin-1 alpha
MCP-1	Monocyte chemoattractant protein-1, chemokine ligand 2
GM-CSF	Granulocyte-macrophage colony-stimulating factor
TNFR2	Tumor necrosis factor receptor 2
IL-6	Interleukin-6
NF-kB	Nuclear factor-kappa B
IL-1β	Interleukin-1 betta
IGF-1	Insulin-like growth factor 1
FGF2	Fibroblast growth factor 2
PIGF	Placental growth factor
