# Supplementary material for: Sex Differences in Circulating Inflammatory, Immune, and Tissue Growth Markers Associated with Fabry Disease-Related Cardiomyopathy
Source: Cells. 2025 Feb 20;14(5):322. doi: 10.3390/cells14050322 (PMC11899294; doi:10.3390/cells14050322)
Supplement: Supplementary file 1 [file cells-14-00322-s001.zip › cells-3451175-supplementary.pdf]

Appendix SA

Appendix SA.1

Supplementary Table S1. Demographics, genotypes, and diagnostic characteristics of patients with Fabry diseases. "#" patients were categorized as a "borderline" due to abnormal EKG. "&" Patients with the severe form of HCM.

No.	Sex M/F	Genotype (Allele1/ Allee2)	Plasma Lyso-Gb-3 ng/ml	Urine Lyso-Gb3 μg/mmol/cr	Therapy (Years)	Age yrs.	Age when therapy started	Age diagnosed with HCM
				**FD patients without HCM**				
1	F	c.718_719del	0.46	8.3	* ERT > 10	25	12	
2	F	C223Y	2.9	70	ERT 5–10	45	37	
3	F	c.966DelC	-	-	ERT < 5	46	44	
4	F	R49P	2.9	<6	ERT > 10	21	10	
5	F	R363 H	0.3	8.6	ERT < 5; CT-2; ERT 2	18	14	
6	F	c1033_1034delTC	1.4	220	* ERT > 10	27	12	
7	F	c.1072_1074del	6.9	151	**naïve**	33		
8	M	422C>T	11	41	ERT > 10	28	15	
9	M	R49P	11	19	* ERT > 10	18	10	
10	M	M296V	0.84	102	ERT 5	36	31	
11	M	A143T	0.39	14	CT > 10	53	38	
12#	F	N215S	0.3	18	ERT- 2; CT- 4	39	34	
13#	F	G328R	5.2	15	* ERT > 10	56	45	
14#	F	C2233Y	4.5	31	ERT < 5	68	66	
15#	M	c.718_719delAA	13	825	ERT < 5	26	24	
16#	M	T41I	0.4	13	CT < 5	58	44	
17#	M	N215S	1.1	57	ERT > 10	42	30	
				**FD patients with HCM**				
18	F	R49P	4.9	40	* ERT 5-10	57	50	50
19	F	R301X	11	63	ERT < 5	41	38	39
20	F	c.718_719delAA	2.9	-	*ERT > 10	46	34	45
21	F	Q279E	2.1	32	CT < 5	46	45	45
22	F	Arg227Gln	2.9	17	ERT < 5	44	44	44
23	F	c1033_1034delTC	2.1	84	CT < 5	21	18	18
24	F	D244N	5.3	96	Mix ERT 5–10, CT	22	13	13
25	F	G325D	2.6	29	ERT > 10	42	27	32
26	F	Gly325Asp	2.7	17	ERT > 10	62	45	61
27	F	A143T	0.3	141	CT < 5	40	38	36
28	F	R118C	0.1	101	naive	59		59
29	M	W277X	18	620	ERT 5–10	24	17	17
30	M	R227Q	15	92	ERT > 10	42	33	41
31	M	V296E	14	1107	*ERT > 10	34	22	34
32	M	G325D	17	177	* ERT > 10	26	12	26
33	M	G325D	5.3	8.7	ERT < 5	20	18	20
34	M	Y134D	6.9	6.4	ERT > 10	25	15	21
35	M	Y207C	12	413	* ERT > 10	53	45	54
36 &	F	N215S	0.6	24	ERT 5–10	65	59	60
37 &	F	c.718_719del	0.3	21	ERT >10	40	22	41
38 &	F	R363H	0.3	21	ERT 5–10	45	48	54
39 &	M	c.966DelC	-	-	ERT 5–10	23	17	-
40 &	M	c1033_1034delTC	77	1033	ERT < 5 y	43	41	42
41 &	M	A143T	0.9	34	ERT < 5	34	31	34
42 &	M	C223Y	20	231	ERT > 10	65	49	59
43 &	M	c.777del	17	33	* ERT > 10	58	43	48
44 &	M	c.717_718del Frameshift	19	1257	* ERT > 10	58	52	58
45 &	M	c.1072_1074del	12	38	ERT 5–10	48	41	44

Appendix SA.2

Supplementary Figure S1. (A) Multivariable analysis between Left ventricular mass (LV mass), Left ventricular posterior wall end diastole and end systole LVPWd, and LV mass/BSA between female and male FD patients with and without HCM. **(B)** Multivariable analysis between left ventricular internal dimension (LVID), end-systolic (LVIDs), and LVPWd in female and male FD patients with and without HCM. **(C) Left:** GM-CSF levels, control vs. FD. Statistical analysis using unpaired T-test and F-test to compare variance. F-test demonstrated a significant difference between control and FD cohorts. * P<0.05. **Right** GM-CSF levels, control vs. FD stratify by sex. F-test * P<0.01.

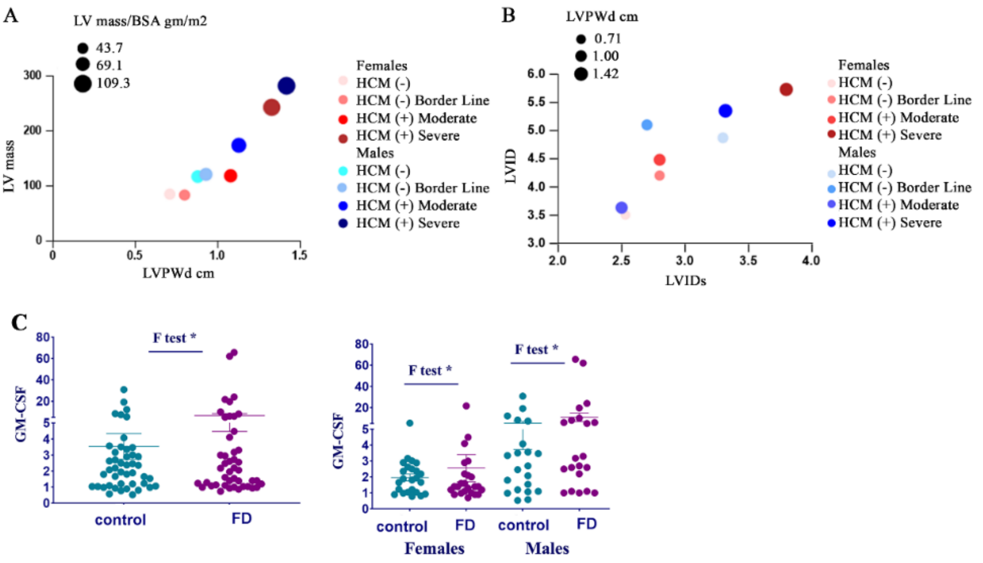

Supplemental Figure 1

Supplementary Table S2. Correlation matrix analysis to compare inflammatory biomarkers and growth factors with plasma or urine Lyso-Gb-3 levels in female and male patients with FD. Additionally, the data was divided into cohorts: "no cardio pathology" HCM(-) and patients with cardiomyopathy HCM(+). The values in the table are for a two-tailed Pearson correlation test with r and P values. Where the r values indicated negative linear correlation (-1 to -0.5), positive linear correlation (0.5 to 1), or absence of linear correlation (between -0.5 to 0.5). P value significant differences p<0.05, Pearson correlation, two tails highlighted with color.

	FGF2	NFkB	Il-6	TNFα	TNFR 2	MCP-1	INF-γ	GM-CSF	PIGF	IGF	IL-10	Il-2	IL-1 α
							**FD female HCM (-)**						
							**Plasma Lyso-Gb-3**						
R	-0.177	0.060	-0.0389	-0.0299	-0.1536	-0.2695	-0.6654	-0.2939	-0.2892	-0.048	-0.094	-0.252	-0.423
P value	0.647	0.876	0.9208	0.9391	0.6932	0.4832	0.0505	0.4427	0.4504	0.901	0.809	0.512	0.255
P summary	ns	ns	ns	ns	ns	ns	ns	ns	ns	ns	ns	ns	ns
							**Urine Lyso-Gb3**						
R	-0.367	-0.496	-0.3165	-0.2052	-0.2545	-0.1873	-0.4382	-0.1069	-0.5331	-0.278	-0.587	-0.540	0.309
P	0.330	0.174	0.4067	0.5964	0.5088	0.6294	0.2381	0.7842	0.1395	0.467	0.095	0.133	0.417
P summary	ns	ns	ns	ns	ns	ns	ns	ns	ns	ns	ns	ns	ns
							**FD female HCM (+)**						
							**Plasma Lyso-Gb3**						
R	-0.16	-0.555	0.1055	0.5583	-0.2015	0.3852	0.1849	0.2339	0.0095	0.245	0.069	0.220	0.217
P	0.584	0.039	0.7198	0.038	0.4898	0.1738	0.527	0.421	0.9741	0.397	0.813	0.449	0.474
P summary	ns	*	ns	*	ns	ns	ns	ns	ns	ns	ns	ns	ns
							**Urine Lyso-Gb3**						
R	0.379	0.174	-0.2832	-0.3584	-0.0366	0.3212	0.4278	0.3801	-0.2637	-0.285	-0.121	0.405	0.567
P	0.201	0.568	0.3484	0.2292	0.9054	0.2846	0.1447	0.2002	0.3841	0.344	0.693	0.169	0.054

P summary	ns	ns	ns	ns	ns	ns	ns	ns	ns	ns	ns	ns	ns
**FD males**													
**FD males HCM (-)**													
**Plasma Lyso-Gb3**													
R	0.208	-0.145	0.3897	-0.3307	-0.4968	0.3294	0.1678	0.6999	0.7456	0.496	-0.046	0.442	0.586
P	0.654	0.755	0.3874	0.4687	0.2567	0.4707	0.7191	0.08	0.0544	0.257	0.920	0.320	0.166
P summary	ns	ns	ns	ns	ns	ns	ns	ns	ns	ns	ns	ns	ns
**Urine Lyso-Gb3**													
R	0.217	0.347	0.2122	-0.4062	-0.0040	0.1535	0.2695	0.2712	-0.0217	0.703	-0.395	-0.086	0.130
P	0.638	0.445	0.6478	0.3659	0.9931	0.7424	0.5589	0.5564	0.963	0.078	0.379	0.852	0.780
P summary	ns	ns	ns	ns	ns	ns	ns	ns	ns	ns	ns	ns	ns
**Male FD HCM (+)**													
**Plasma Lyso-Gb3**													
R	0.128	-0.254	0.0011	-0.138	-0.0508	0.0607	-0.1537	-0.2811	0.0059	0.108	-0.076	-0.081	-0.272
P	0.675	0.401	0.9971	0.6531	0.8689	0.8436	0.6335	0.3522	0.9845	0.725	0.804	0.792	0.368
P summary	ns	ns	ns	ns	ns	ns	ns	ns	ns	ns	ns	ns	ns
**Urine Lyso-Gb-3**													
R	0.356	-0.42	-0.3921	-0.0353	0.3133	-0.1511	-0.3278	-0.3965	0.648	-0.038	-0.198	-0.249	-0.437
P	0.231	0.153	0.1851	0.9087	0.2973	0.6223	0.2982	0.1799	0.0166	0.900	0.515	0.410	0.134
P summary	ns	ns	ns	ns	ns	ns	ns	ns	*	ns	ns	ns	ns

Supplementary Table S3. The Pearson correlation analysis matrix assesses the relationship between two variables in a data set. The table displays the P-value for the two tails Pearson correlation. P<0.05 significant linear correlation.

Female HCM(-)															
	FGF2	NFkB	Il-6	TNFα	TNFR2	MCP-1	INFγ	VEGF	GM-CSF	PIGF	TGFβ	Active TGFβ	IGF-1	IL-10	IL-2
FGF2															
NFkB	0.346														
Il-6	0.914	0.393													
TNFα	0.103	0.994	0.729												
TNFR2	0.712	0.595	0.713	0.573											
MCP-1	0.984	0.783	0.914	0.997	0.011										
INFγ	0.908	0.763	0.726	0.589	0.013	0.025									
VEGF	0.386	0.456	0.021	0.359	0.753	0.763	0.410								
GM-CSF	0.563	0.669	0.395	0.851	0.077	0.006	0.245	0.219							
PIGF	0.699	0.402	0.235	0.761	0.455	0.546	0.386	0.351	0.275						
TGFβ	0.297	0.077	0.437	0.679	0.675	0.934	0.450	0.101	0.313	0.123					
Active TGFβ	0.165	0.371	0.447	0.872	0.070	0.327	0.463	0.113	0.048	0.665	0.483				
IGF-1	0.459	0.711	0.980	0.942	0.260	0.601	0.970	0.700	0.667	0.210	0.640	0.094			
IL-10	0.806	0.018	0.858	0.452	0.331	0.183	0.798	0.622	0.286	0.813	0.262	0.237	0.382		
IL-2	0.929	0.620	0.927	0.246	0.819	0.559	0.824	0.604	0.618	0.407	0.431	0.457	0.621	0.116	
IL-1α	0.884	0.413	0.014	0.819	0.964	0.967	0.734	0.001	0.124	0.128	0.034	0.149	0.871	0.884	0.196
Female HCM+															
	FGF2	NFkB	Il-6	TNFα	TNFR2	MCP-1	INFγ	VEGF	GM-CSF	PIGF	TGFβ	Active TGFβ	IGF-1	IL-10	IL-2
FGF2	0.8101														
NFkB	0.8101														
Il-6	0.7335	0.5453													
TNFα	0.8852	0.0862	0.5863												
TNFR2	0.9876	0.7533	0.7311	0.3168											
MCP-1	0.9830	0.1425	0.6202	0.9516	0.2588										
INFγ	0.4714	0.3048	0.3361	0.9882	0.8905	0.7165									
VEGF	0.6443	0.4265	0.3773	0.7180	0.2797	0.0037	0.4284								
GM-CSF	0.6264	0.4211	0.4519	0.6930	0.2872	0.0041	0.5603	0.0000							
PIGF	0.9165	0.8111	0.0488	0.7856	0.4655	0.1183	0.0860	0.2401	0.1993						
TGFβ	0.8663	0.1537	0.9134	0.4475	0.3257	0.3814	0.5652	0.1547	0.2245	0.8023					
Active TGFβ	0.3263	0.5377	0.5194	0.1420	0.7297	0.1444	0.1660	0.0200	0.0238	0.0433	0.0201				
IGF-1	0.8164	0.0827	0.9480	0.0182	0.5194	0.5758	0.7835	0.4523	0.3942	0.5965	0.1132	0.1964			
IL-10	0.3877	0.4497	0.5403	0.9600	0.6597	0.8775	0.1280	0.2653	0.2744	0.9057	0.9968	0.2781	0.7067		
IL-2	0.8926	0.4795	0.1725	0.8096	0.2562	0.0090	0.7365	0.0000	0.0000	0.3055	0.3914	0.1148	0.2855	0.3084	
IL-1α	0.8741	0.4667	0.3006	0.6249	0.4254	0.0037	0.6396	0.0001	0.0004	0.2489	0.1571	0.0197	0.7718	0.2816	0.0001
Male HCM(-)															
	FGF2	NFkB	Il-6	TNFα	TNFR2	MCP-1	INFγ	VEGF	GM-CSF	PIGF	TGFβ	Active TGFβ	IGF-1	IL-10	IL-2
FGF2															
NFkB	0.556														
Il-6	0.228	0.293													

TNFα	0.194	0.130	0.607												
TNFR2	0.424	0.990	0.285	0.822											
MCP-1	0.172	0.890	0.066	0.823	0.621										
INFγ	0.179	0.342	0.825	0.981	0.701	0.143									
VEGF	0.726	0.965	0.262	0.907	0.427	0.093	0.194								
GM-CSF	0.470	0.800	0.233	0.706	0.408	0.043	0.152	0.001							
PIGF	0.516	0.275	0.141	0.803	0.447	0.151	0.588	0.081	0.024						
TGFβ	0.230	0.717	0.993	0.240	0.120	0.790	0.665	0.475	0.535	0.192					
Active TGFβ	0.785	0.590	0.645	0.122	0.870	0.371	0.974	0.407	0.279	0.537	0.602				
IGF-1	0.462	0.595	0.559	0.578	0.903	0.552	0.449	0.734	0.862	0.971	0.357	0.306			
IL-10	0.451	0.317	0.245	0.099	0.692	0.208	0.913	0.505	0.305	0.346	0.562	0.004	0.360		
IL-2	0.078	0.150	0.072	0.251	0.828	0.117	0.368	0.143	0.048	0.014	0.790	0.468	0.905	0.239	
IL-1α	0.415	0.759	0.265	0.535	0.437	0.038	0.169	0.006	0.000	0.032	0.685	0.149	0.628	0.173	0.046
Male HCM+															
	FGF2	NFkB	Il-6	TNFα	TNFR2	MCP-1	INFγ	VEGF	GM-CSF	PIGF	TGFβ	Active TGFβ	IGF-1	IL-10	IL-2
FGF2															
NFkB	0.578														
Il-6	0.134	0.438													
TNFα	0.836	0.828	0.362												
TNFR2	0.264	0.796	0.752	0.669											
MCP-1	0.970	0.003	0.426	0.280	0.737										
INFγ	0.495	0.000	0.939	0.251	0.872	0.000									
VEGF	0.697	0.004	0.845	0.408	0.998	0.002	0.000								
GM-CSF	0.935	0.001	0.975	0.103	0.745	0.003	0.000	0.011							
PIGF	0.081	0.272	0.226	0.728	0.032	0.744	0.355	0.585	0.378						
TGFβ	0.836	0.386	0.546	0.222	0.410	0.672	0.975	0.906	0.796	0.842					
Active TGFβ	0.229	0.984	0.855	0.002	0.894	0.582	0.734	0.810	0.134	0.663	0.326				
IGF-1	0.269	0.506	0.054	0.006	0.298	0.654	0.967	0.941	0.925	0.367	0.174	0.263			
IL-10	0.664	0.004	0.723	0.323	0.450	0.000	0.000	0.000	0.015	0.748	0.969	0.838	0.949		
IL-2	0.710	0.007	0.896	0.175	0.859	0.001	0.000	0.000	0.010	0.501	0.825	0.845	0.722	0.000	
IL-1α	0.998	0.114	0.876	0.208	0.626	0.167	0.054	0.003	0.022	0.909	0.617	0.522	0.551	0.082	0.028

Appendix SB

All appendix sections must be cited in the main text. In the appendices, Figures, Tables, etc. should be labeled starting with "A"—e.g., Figure A1, Figure A2, etc.
